# Synergistic antimicrobial potential of EGCG and fosfomycin against biofilms associated with endodontic infections

**DOI:** 10.1590/1678-7757-2022-0282

**Published:** 2023-03-09

**Authors:** Cristiane DUQUE, Amanda Caselato Andolfatto SOUZA, Kelly Limi AIDA, Jesse Augusto PEREIRA, Karina Sampaio CAIAFFA, Vanessa Rodrigues dos SANTOS, Leopoldo COSME-SILVA, Anuradha PRAKKI

**Affiliations:** 1 Universidade Estadual Paulista Faculdade de Odontologia de Araçatuba Departamento de Odontologia Preventiva e Restauradora Araçatuba SP Brasil Universidade Estadual Paulista (UNESP), Faculdade de Odontologia de Araçatuba, Departamento de Odontologia Preventiva e Restauradora, Araçatuba, SP, Brasil.; 2 Universidade Federal de Alagoas Departamento de Odontologia, Área de Endodontia Alagoas CE Brasil Universidade Federal de Alagoas, Departamento de Odontologia, Área de Endodontia, Alagoas, CE, Brasil.; 3 University of Toronto Faculty of Dentistry Dental Research Institute Toronto ON Canada University of Toronto, Faculty of Dentistry, Dental Research Institute, Toronto, ON, Canada.

**Keywords:** Epigallocatechin gallate, Fosfomycin, Drug synergism, Biofilms, Cytotoxicity tests

## Abstract

**Objective:**

This study aimed to evaluate the cytotoxicity and synergistic effect of epigallocatechin gallate (EGCG) and fosfomycin (FOSFO) on biofilms of oral bacteria associated with endodontic infections.

**Methodology:**

This study determined minimum inhibitory and bactericidal concentration (MIC/MBC) and fractionated inhibitory concentration (FIC) of EGCG and FOSFO against *Enterococcus faecalis*, *Actinomyces israelii*, *Streptococcus mutans*, and *Fusobacterium nucleatum*. Monospecies and multispecies biofilms with those bacteria formed in polystyrene microplates and in radicular dentin blocks of bovine teeth were treated with the compounds and control chlorhexidine (CHX) and evaluated by bacterial counts and microscopy analysis. Toxicity effect of the compounds was determined on fibroblasts culture by methyl tetrazolium assays.

**Results:**

The combination of EGCG + FOSFO demonstrated synergism against all bacterial species, with an FIC index ranging from 0.35 to 0.5. At the MIC/FIC concentrations, EGCG, FOSFO, and EGCG+FOSFO were not toxic to fibroblasts. EGCG+FOSFO significantly reduced monospecies biofilms of *E. faecalis* and *A. israelli*, whereas *S. mutans* and *F. nucleatum *biofilms were eliminated by all compounds. Scanning electron microscopy of multispecies biofilms treated with EGCG, EGCG+FOSFO, and CHX at 100x MIC showed evident biofilm disorganization and substantial reduction of extracellular matrix. Confocal microscopy observed a significant reduction of multispecies biofilms formed in dentin tubules with 84.85%, 78.49%, and 50.6% of dead cells for EGCG+FOSFO, EGCG, and CHX at 100x MIC, respectively.

**Conclusion:**

EGCG and fosfomycin showed a synergistic effect against biofilms of oral pathogens related to root canal infections without causing cytotoxicity.

## Introduction

The endodontic treatment aims to disinfect the root canal system and obturate the space to eliminate any residual microorganisms and prevent reinfection in teeth with apical periodontitis.^1^ New technologies, such as nickel-titanium files and rotary instrumentation, have simplified instrumentation procedures, reduced treatment time, and increased the predictability of endodontic treatments. These procedures favor the retention of essential irrigants, which are necessary for their proper activation and dispersion throughout the endodontic system, resulting in better cleaning, especially in the apical portion, better treatment outcome.^2,3^ Although a significant reduction in the number of bacteria can be achieved by cleaning, shaping, and irrigating the canal during endodontic procedures,^1-4,5^ it is not possible to attain complete disinfection of the root canal system by any of the available irrigants due to its anatomical complexity and bacterial resistance.^1,4,5^

Some microorganisms are resistant to antimicrobial treatment and can survive in the root canal after chemo-mechanical preparation. The most common bacteria are the Gram-negative anaerobic bacteria, such as *Fusobacterium nucleatum*, *Prevotella* spp., and *Campylobacter rectus,* and some Gram-positive bacteria, such as *Streptococcus, Lactobacillus, Actinomyces, *and* Enteroccocus faecalis*, among others.^6^ Supplementary approaches, such as intracanal medications, have been used to eliminate surviving microorganisms inside dentin tubules and inhibit bacterial growth.^7^ Calcium hydroxide is widely used as intracanal medication due to its bactericidal action and ability to neutralize the pH of remaining pulp tissues. However, calcium hydroxide may not be effective against all bacteria found in the root canal and requires multiple sessions to increase its antimicrobial action.^7,8^ Triple antibiotic paste (TAP), a combination of metronidazole, ciprofloxacin, and minocycline, has been used as an intracanal dressing to eliminate pathogens in teeth with pulp necrosis and incomplete root formation in regenerative endodontic protocols. However, the use of TAP has generated some concerns regarding bacterial resistance, crown discoloration, and possible allergic reactions.^9^

New therapies have emerged to improve the antimicrobial efficiency and to enhance the penetration of irrigants into the complex root canal anatomy. These include antimicrobial photodynamic therapy (aPDT), laser-activated irrigation, and the use of sonic and ultrasonics activation.^10^ Additionally, natural products, such as polyphenols, have been explored as alternative endodontic medicaments due to their wide range of biological properties, such as antimicrobial, antioxidant, anti-inflammatory, and anti-tumoral activities, among others.^11^ Polyphenols are classified into four categories based on the presence of phenolic groups and structural elements: flavonoids, stilbenes, lignans, and phenolic acids.^11^ Epigallocatechin-3-gallate (EGCG) is a major flavonoid compound extracted from green tea leaves, which represents approximately 59% of the total catechins.^11^ In addition to its anti-oxidative, anti-tumor, and anti-inflammatory properties, EGCG has demonstrated effectiveness against both Gram-positive and Gram-negative bacteria,^12,13^ including multidrug-resistant bacteria such as *Pseudomonas aeruginosa *and *Escherichia coli*.^14^ EGCG has also been shown to be efficacious against *Enterococcus faecalis*, both in planktonic cells and biofilm,^15^ and it is also efficacious at inhibiting the adhesion of *Streptococcus mutans* in a dose-dependent manner.^16^ Furthermore, EGCG also demonstrated potent inhibition of metalloproteinases (MMP), mainly MMP-2 and MMP-9,^17^ which are host-derived enzymes associated with the self-degradation of dentin collagen and the progression of dental caries, periodontitis, and apical periodontitis.^17,18^

Studies have demonstrated that low concentrations of EGCG can enhance antimicrobial activity of β-lactam antibiotics against multidrug-resistant bacteria by attacking the same site on cell walls as the antibiotics, specifically the peptidoglycan.^19^ In addition, the combination of antibiotics with EGCG has been shown to increase the antibacterial effect on oral biofilms.^20^ The antibiotic fosfomycin is a phosphonic acid derivative with wide-spectrum activity against several bacteria, including multidrug-resistant bacteria, since it inhibits an enzyme-catalyzed reaction in the first step of bacteria cell wall synthesis.^21-22^ Synergistic effect of fosfomycin can be observed when it is combined with other antimicrobial agents that present different mechanism of action, thereby allowing for reduced dosages and lower toxicity.^21^

This study aimed to evaluate the cytotoxicity and antibacterial effect of EGCG and fosfomycin, alone and in combination, on endodontic bacteria in both planktonic and biofilm conditions. Due to the large number of species involved in endodontic infections and the need for a broad-spectrum medicament to be used in root canals between appointments, the combinations of these drugs could be promising in controlling infection and inflammation before root canal obturation. We hypothesized that the combined use of EGCG and fosfomycin will present superior antibacterial effect without affecting cell viability. This novel combination could be used as an effective bioactive agent for both irrigants and intracanal medication in endodontic treatments.

## Methodology

### Preparation of the compounds

The compounds tested were fosfomycin sodium (FOSFO; # 34089, Sigma-Aldrich, St Louis, MO, USA), epigallocatechin gallate (EGCG; #E4143, Sigma Aldrich), and chlorhexidine digluconate (CHX) (Pharmacia Manipullis, Araçatuba, SP, Brazil) as the control. The compounds were weighed with an analytical scale (OHAUS Adventurer, Parsippany, NY, USA) at 4 mg/mL (FOSFO, EGCG) or 20 mg/mL (CHX) and dissolved in sterile deionized water. All solutions were filtered using 0.2µm syringe filters and stored at -20°C (FOSFO, EGCG) or 4°C (CHX), following the manufacturer’s recommendations.

### Bacterial strains and culture conditions

The following standard strains were used in the antimicrobial/antibiofilm assays: *Enterococcus faecalis *(ATCC 51299),* Actinomyces israelii *(ATCC 12102), *Streptococcus mutans *(ATCC 25175*), *and *Fusobacterium nucleatum* (ATCC 25586), which were kindly donated by Fundação Oswaldo Cruz (FIOCRUZ, RJ, Brazil). The culture media used for each bacterial species were as follows: Mitis Agar Salivarius Agar (Difco, Kansas City, MO, USA) with 0.2 U/mL bacitracin for *S. mutans*, and Brain Heart Infusion Agar – BHIA (Difco) for *A. israelii *and *E. faecalis*. For *F. nucleatum*, the medium was supplemented BHIA containing 5 mg/L hemin, 5 mg/mL menadione, and 5% defibrinated sheep blood. All microorganisms were incubated at 37°C in an atmosphere of 5% CO_2_, except for *F. nucleatum*, which was cultivated in anaerobic conditions, using jars and gas-pak sachets at 37°C (Anaerogen, Thermo Fisher, Waltham, MA, USA). Each analysis described below was performed in triplicate on three different days.^23^

### Determination of Minimal Inhibitory Concentration (MIC) and Minimum Bactericidal Concentration (MBC)

MIC was determined using the microdilution method, based on the criteria by the Clinical and Laboratory Standard Institute M7-A9 – CLSI.^24^ Microbial suspensions were incubated in either Mili-Q water or CHX as negative and positive controls, respectively. MIC was defined as the lowest concentration of the compound (FOSFO, EGCG, or CHX), which presented no visible growth. The media containing this MIC concentration and two higher concentrations were serially diluted and plated on Miller Hinton agar medium and incubated at 37°C for 48 h to obtain MBC, which was defined as the minimal concentration of the compound required to reduce more than 99.9% of bacteria. The colony forming unit (CFU)/mL was counted using a binomial stereomicroscope.^23^

### Determination of Fractional Inhibitory Concentration (FIC)

The synergistic effects of EGCG and fosfomycin were determined by the fractional inhibitory concentration (FIC) using the microdilution checkerboard method. The combination value was derived from the highest dilution of the non-growth antimicrobial combination. The FIC index was determined by the equation: FIC index = FIC A + FIC B ((MIC of antimicrobial A in combination/MIC of A alone) + (MIC of antimicrobial B in combination/MIC of B alone)). Synergy was defined when the FIC index was ≤0.5; FIC additive when> 0.5-4; and antagonistic when FIC was> 4.0.^25^

### Cytotoxicity assays

Cytotoxicity assays were conducted following Caiaffa, et al.^23^ (2017). Briefly, L929 mouse fibroblast cell line (ATCC CC-1) was grown in Dulbecco’s modified Eagle’s medium (DMEM; Gibco, Grand Island, NY, USA) supplemented with 10% fetal bovine serum (FBS; Gibco) and 100 IU/mL of penicillin, 100 μg/mL of streptomycin, and 2 mmol/mL of glutamine (Gibco) in a humidified incubator under 5% CO_2_ and 95% air at 37°C (Thermo Fisher). After 24 h growth in 96-well microplates, cells were stimulated with FOSFO, EGCG, CHX, and different combinations of EGCG and FOSFO, based on microbiological assays. The cell metabolism was assessed by methyl tetrazolium (MTT) assays after 24 h of exposure. The means were calculated for the groups and transformed into percentage cell viability in relation to the negative control (DMEM), which was defined as having 100% cell metabolism.

### Antibiofilm activity

#### Monospecies biofilm assays in polystyrene microplates

The assays were conducted following Massunari, et al.^26^ (2017), with monospecies biofilms of *Enterococcus faecalis, Actinomyces israelii*, *Streptococcus mutans*, and *Fusobacterium nucleatum*. Briefly, in sterile U-shaped bottom polystyrene 96-well microplates, a pretreatment with 200 μL/well of artificial saliva (800 mL of deionized water, 1.6 g of yeast extract, 4 g of peptone, 0.28 g of NaCl, 1.6 g of glucose or 3.2 g of sucrose, 0.16 g of CaCl_2_, 0.16 g of KCl, and 0.8 g of mucin) was applied for 4 h at 37°C in a 5% CO_2_ atmosphere (coating phase). After the incubation period, the saliva was removed and 200 μL of each bacterial culture were inserted in each well (approximately 1-5 × 10⁶ CFU/mL) in supplemented BHI broth (5 mg/L hemin, 5 mg/mL menadione, and 5% defibrinated sheep blood) containing 0.5% sucrose (for* S. mutans*) or 1% glucose (other bacterial strains). Plates were incubated at 37°C in 5% CO_2_ for 48 h, except for* F. nucleatum, *which was incubated for 72 h in an anaerobic condition. After these periods, the culture medium was removed and the wells were washed with sterile saline (0.9% NaCl) for subsequent addition of 150 μL in each well of the compounds EGCG at 10× MIC, EGCG +FOSFO at 10× FIC, and CHX at 10× or 100× MIC for each bacterial species. The microplates were incubated for 24 h under the same conditions as previously described. Next, the treatments were removed, the biofilms were scraped off with sterile plastic cell scrapers and saline solution, serially diluted and plated in BHIA. After 48h of incubation, CFU/mL was determined.

#### Multispecies biofilm assays in polystyrene microplates and SEM analysis

The multispecies biofilm assays were conducted following the same steps described previously for monospecies biofilms; however, all bacteria (*E. faecalis*, *A. israelii*, *S. mutans,* and *F. nucleatum*) were mixed in equal aliquots at the same concentration (1-5× 10^3^ CFU/mL) in supplemented BHI broth containing 1% glucose. The inoculum was inserted in 24 wells microplates and incubated for one week in anaerobic conditions. After that, biofilms were washed twice with sterile saline solution and the following treatments were performed: 1) EGCG at 10× the highest MIC (2.5 mg/mL) and 2) EGCG + FOSFO at 10× the highest FIC (0.625mg/mL EGCG and 0.078 mg/mL FOSFO). For controls: 3) CHX at 10× the highest MIC - 0.05 mg/mL and 4) CHX at 100× the highest MIC - 0.5 mg/mL. The plates were incubated for 48 h at 37°C in anaerobic conditions. After scraping of biofilms, aliquots from all wells were resuspended, serially diluted, and plated on BHI agar. Then, the plates were incubated for 48 h for further counting of CFU/mL. Parallel experiments with multispecies biofilms were conducted in coverslips for scanning electron microscopy (SEM) analysis. However, the biofilms were dehydrated by washing in a series of ethanol (70% for 10 min, 95% for 10 min, and 100% for 20 min) and air-dried in a desiccator. Afterwards, coverslips were mounted into aluminum stubs, sputter coated with gold, and analyzed in a scanning electron microscope (Leo, Cambridge, MA, USA).^27^

#### Multispecies biofilm in dentin tubules of bovine roots and CLSM analysis

Biofilm assays for confocal laser scanning microscopy (CLSM) analysis were conducted with all bacteria (*E. faecalis*, *A. israelii*, *S. mutans,* and *F. nucleatum*) mixed in equal aliquots at the same concentration (1-5× 10^3^ CFU/mL) in BHI broth containing 1% glucose. The experiments were conducted following Ma, et al.^28^ (2011) and Caiaffa, et al.^23^(2017). Briefly, 4 mm cylindrical dentin blocks (n=6/group) from bovine incisor roots (Ethics Committee on the Use of Animal approval, protocol #01194-2017) were cut using a 0.6 mm diamond saw at 1000 rpm under water cooling (Isomet 5000, Buehler Ltda, Lake Bluff, IL). After enlarging the root canals with a Gates Glidden (#6, 1.5 mm in diameter) drill, dentin blocks were fractured into two semi cylindrical halves (3×3×2 mm) using a using a handpiece diamond disc. After that, they were autoclaved and washed in 17% EDTA solution for 3 min and, then, in distilled water for 5 minutes in ultrasonic bath. After sterilization in autoclave, each dentin block was fixed in a microtube and infected with 500 μL of bacterial mixture suspension by sequential centrifugation for 5 minutes. Dentin blocks were incubated individually in 48-well plates in supplemented BHI broth for 14 days, replacing the culture medium every 72 h. After this period, the blocks were washed twice with saline solution and transferred, under aseptic conditions, to a new plate and exposed to EGCG at 10× the highest MIC (2.5 mg/mL); EGCG + FOSFO at 10× the highest FIC (0.625mg/mL EGCG and 0.078 mg/mL FOSFO); and controls (CHX at 10× the highest MIC - 0.05 mg/mL and 100× the highest MIC - 0.5 mg/mL) for 48 h in static conditions. Subsequently, the dentin blocks were washed again twice, cut into transverse slices of 1 mm thickness, and stained with 100 µL of fluorescent LIVE/DEAD BacLight Bacterial Viability stain (L13152, Molecular Probes, Eugene, OR). Two additional uninfected specimens were stained using the same protocol as the negative controls. The mounted specimens were observed using a 63× NA 1.4 oil immersion lens and CLSM images were acquired using the software LAS AF (Leica Mic-systems). Each 2D (two-dimensional) image was obtained by the max projection of the Z stack. The ratio of red fluorescence to green-and-red fluorescence indicated the proportion of dead cells to total cells for each antimicrobial agent, measured using Image J software (Rasband, W.S., Image J, U. S. National Institutes of Health, Bethesda, Maryland, USA, https://imagej.nih.gov/ij/, 1997-2016).

## Statistical analyses

Data from cytocompatibility and microbiological assays were expressed in means/standard deviation and subjected to ANOVA and Tukey’s tests, considering each antimicrobial agent separately. Bacterial counts (from biofilm assays) were transformed in log (CFU+1/mL) due to the variability of the data. SPSS 19.0 software (SPSS Inc., Chicago, IL, USA) was used to run the statistical analysis considering p<0.05.

## Results

### Antimicrobial activity

Table 1 shows values of minimum inhibitory concentration (MIC), minimum bactericidal concentration (MBC), and fractional inhibitory concentration (FIC) values for EGCG, Fosfomycin (FOSFO), and the positive control Chlorhexidine (CHX), which presented the lowest MIC/MBC values against all bacteria tested. The MIC and MBC values ranged from 0.031 to 0.25 mg/mL for EGCG and 0.00097 to 0.062 mg/mL for FOSFO. The FIC values ranged from 0.0039 to 0.062 mg/mL for EGCG and 0.0001 to 0.015 mg/mL for FOSFO. The combination of EGCG and FOSFO showed synergism against all bacterial species, with an FIC index ranging from 0.35 to 0.5 mg/mL. Among the bacteria evaluated, *A. israelii* and *F. nucleatum *were the most sensitive to both EGCG and FOSFO alone, as well as their combination. *E. faecalis *was inhibited by EGCG alone at 0.25 and 0.5 mg/mL, respectively. However, the combination of EGCG concentration at 0.062 mg/mL and FOSFO at 0.0078 mg/mL was more effective in inhibiting *E. faecalis*. *S. mutans *was eliminated with 0.5 mg/mL EGCG or 0.125 mg/mL FOSFO, whereas their combination at lower concentrations of 0.062 and 0.015 mg/mL, respectively, was also effective.

**Table 1:** MIC, MBC, and FIC values (mg/mL) for EGCG, Fosfomycin, and their combination

Bacteria	Compounds	MIC	MBC	FIC (EGCG+Fosfomycin)
*E. faecalis*	EGCG Fosfomycin Chlorhexidine	0.25 0.031 0.0078	0.5 0.062 0.0078	FIC=0.5 EGCG 0.062 + Fosfomycin 0.0078
*A. israelii*	EGCG Fosfomycin Chlorhexidine	0.031 0.0019 0.0019	0.062 0.0078 0.0019	FIC=0.37 EGCG 0.0039 + Fosfomycin 0.00049
*S. mutans*	EGCG Fosfomycin Chlorhexidine	0.25 0.0625 0.00097	0.5 0.125 0.0019	FIC=0.5 EGCG 0.062 + Fosfomycin 0.015
*F. nucleatum*	EGCG Fosfomycin Chlorhexidine	0.062 0.00097 0.00078	0.062 0.00097 0.00078	FIC=0.35 EGCG 0.015 + Fosfomycin 0.0001

### Fibroblast viability

**Figure 1:**
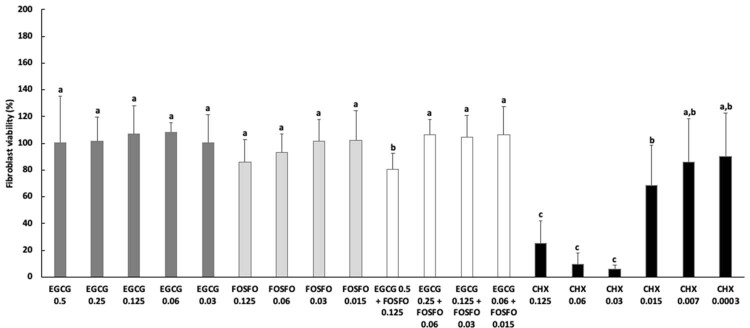
Cell viability (%) after 24 h of exposure to different concentrations of EGCG, Fosfomycin (FOSFO), EGCG with FOSFO combinations at different concentrations, and control chlorhexidine (CHX). DMEM was considered as 100% cell metabolism. ^a^ Different letters show statistical difference among the antimicrobial groups and concentrations, according to ANOVA and Tukey’s test (p<0.05).

The cytotoxicity effect of EGCG, FOSFO, EGCG+FOSFO, and CHX was evaluated on fibroblastic cell culture (Figure 1). Although the highest concentration of EGCG and FOSFO in combination showed the lowest results in relation to cell viability, this remained above 80%, showing cytocompatibility. CHX was the most cytotoxic compound tested in this study. Its cytotoxic effect was reduced only in concentrations below 0.015 mg/mL.

### Antibiofilm effect of the compounds

#### Monospecies biofilms

**Figure 2:**
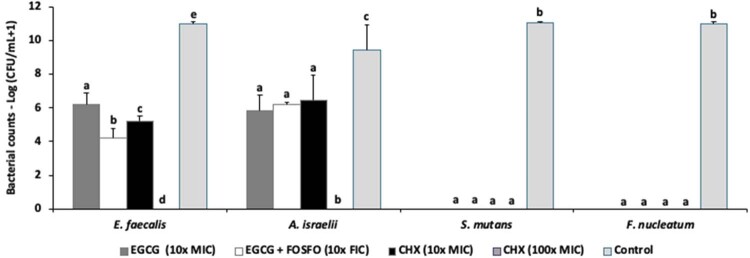
Effect of EGCG alone (at 10× MIC for each bacterium), EGCG in combination with fosfomycin (at 10x FIC for each bacterium), and control chlorhexidine (10× and 100× MIC for each bacterium) on biofilms of *E. faecalis*, *A. israelii*, *S. mutans*, and *F. nucleatum.* ^a^ Different letters show statistical difference among the antimicrobial groups, according to ANOVA and Tukey’s test (p<0.05). Values expressed as means (standard deviation - SD) of bacterial counts in Log (CFU/mL +1). CFU – colony forming units; FOSFO – fosfomycin; CHX – chlorhexidine.

The biofilm of *E. faecalis *was significantly reduced by EGCG + FOSFO at 10x FIC (-6.2 log), followed by CHX at 10× MIC (-5.8 log) and EGCG at 10× MIC (-4.8 log), compared to the control. *A. israelii *biofilms were also reduced by all antimicrobial agents without statistical difference among them (reduction of 2.98 to 3.59 log from the control). *S. mutans *and *F. nucleatum *biofilms were eliminated by all compounds. CHX at 100x MIC eliminated all monospecies biofilms tested (Figure 2).

#### Multispecies biofilms and SEM analysis

**Figure 3:**
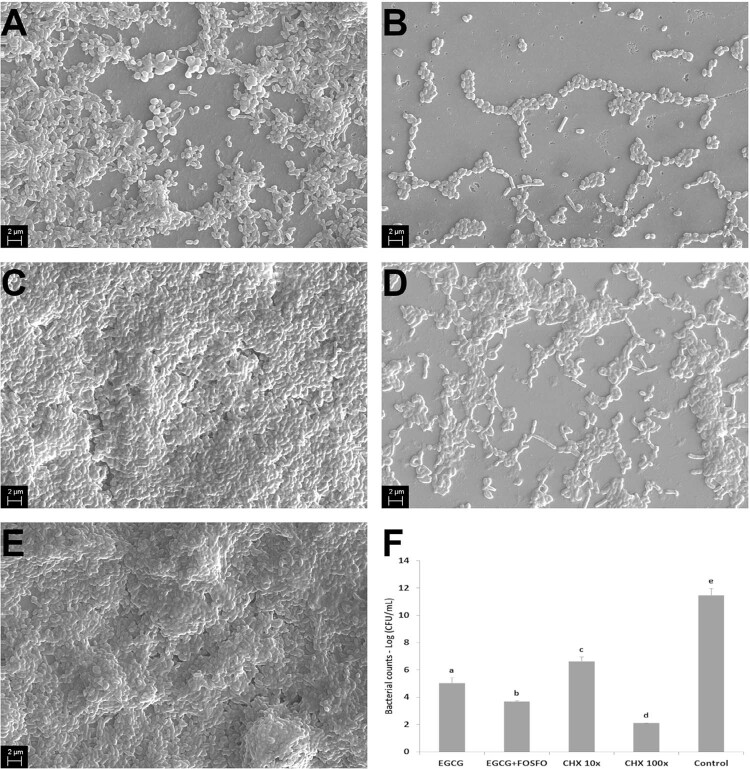
Representative scanning electron microscopy images of 14-day multispecies biofilms under 1000× magnification. Biofilms were treated for 48 hours with A – EGCG (at 2.5 mg/mL); B – EGCG (at 0.62 mg/mL) and FOSFO (at 0.078 mg/mL); C – CHX at 0.05 mg/mL; D – CHX at 0.5 mg/mL; and E – Control group showing the bacterial growth without antimicrobial agents. The F figure represents mean (SD) of the bacterial counts detected after 48 h of the biofilm treatment with the compounds. ^a^ Different letters show statistical difference among the antimicrobial groups, according to ANOVA and Tukey’s test (p<0.05).

Figure 3 (A-G) shows representative images of scanning electron microscopy of multispecies biofilms with *E. faecalis, A. israelli, S. mutans, and F. nucleatum *after treatment with the antimicrobial agents tested. Evident biofilm disorganization and areas of substantial reduction of extracellular matrix and bacterial presence are observed in Figures 3A, B, and D when multispecies biofilms were treated with EGCG, EGCG +FOSFO, and CHX 100× MIC. Bacteria co-aggregated and incorporated in an extracellular polymeric matrix, which can be seen in Figure 3C (CHX 10×) and Figure 3E (control without the antimicrobial agents). Figure 3F presents the effect of all compounds on bacterial counts (in log CFU/mL) in the multispecies biofilms, confirming that CHX 100×, EGCG+FOSFO, and EGCG were the most effective treatments, reducing 9.35 log, 7.79 log, and 6.43 log UFC/mL, respectively.

#### Multispecies biofilms in dentin tubules and CLSM analysis

**Figure 4:**
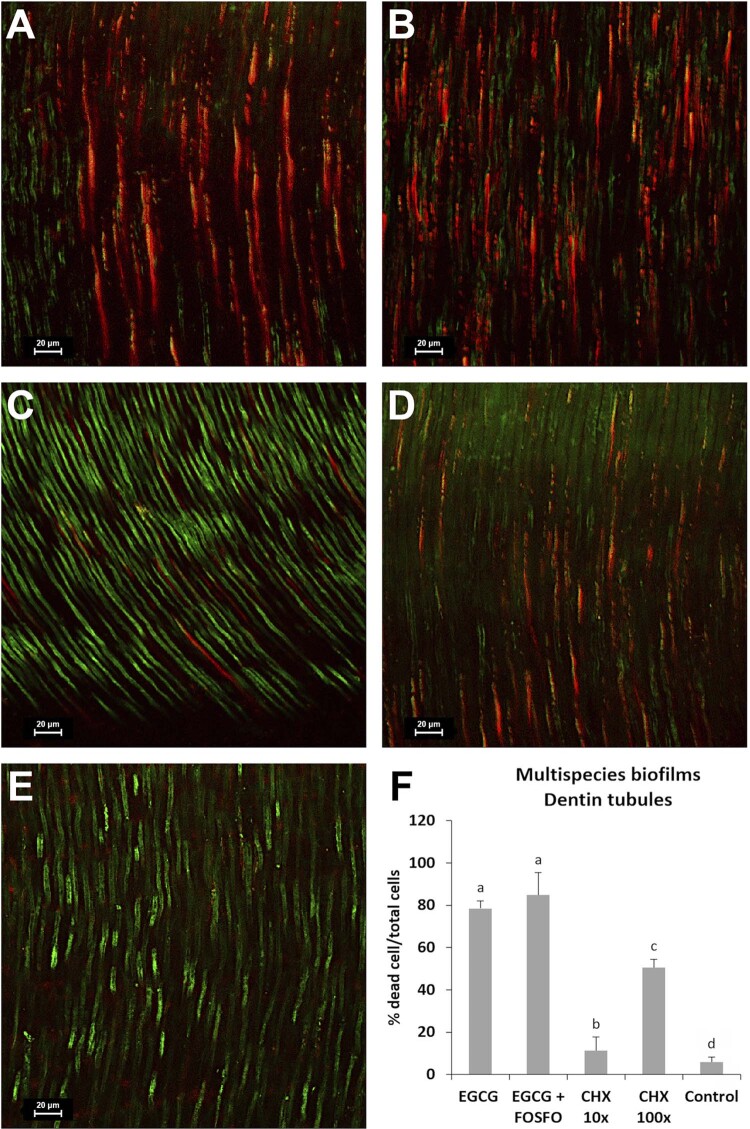
Representative confocal microscopy images of bovine root dentin specimens contaminated for 14 days with multispecies biofilms and treated for 48 hours with the following groups with A – EGCG (at 2.5 mg/mL); B – EGCG (at 0.62 mg/mL) + FOSFO (at 0.078 mg/mL); C – CHX at 0.05 mg/mL; D – CHX at 0.5 mg/mL; and E - Control group showing the bacterial growth without antimicrobial agents. The F figure represents mean (SD) of the percentages of dead cells of multispecies biofilms after 48h of treatment with the compounds. Bacterial counts were obtained by Image J analysis. ^a^ Different letters show statistical difference among the antimicrobial groups, according to ANOVA and Tukey’s test (p<0.05).

The effect of EGCG+FOSFO and CHX was also observed on multispecies biofilm formed in dentinal tubules of bovine teeth, as shown in Figure 4. Figures 4A-E shows representative images of confocal microscopy, demonstrating superior bactericidal effect of the EGCG, EGCG + FOSFO, and CHX 100×. There is no statistical difference between EGCG and EGCG+FOSFO, however, the concentrations of EGCG (0.62 mg/mL) in combination with FOSFO were much lower than EGCG alone (2.5 mg/mL). A significant reduction in the bacterial counts can be observed in Figure 4F for all groups, with 84.85%, 78.49%, and 50.6% of dead cells in dentinal tubules for EGCG+FOSFO at 10× FIC, EGCG 10× MIC, and CHX at 100× MIC, respectively.

## Discussion

This study aimed to evaluate the cytotoxicity and effect of combined EGCG and fosfomycin on bacteria of endodontic interest in both planktonic and biofilm conditions. The findings support the hypothesis that the combination of EGCG and FOSFO presents a better antibacterial effect than when used individually without affecting cell viability. The combination of conventional antibiotics with natural compounds, such as EGCG, has emerged as a strategy to address bacterial resistance and reduce the antimicrobial concentration of natural compounds, which are expensive to synthesize and purify.^29^ These plant-derived polyphenols, such as EGCG, have been receiving worldwide attention due to their antimicrobial, antioxidant, anti-inflammatory, antiproteolytic, and anti-tumoral activities.^11^

In this study, *A. israelii* and *F. nucleatum* were found to be the most sensitive to EGCG, FOSFO or their combination, whereas *E. faecalis *was the most resistant bacteria. Some studies have also evaluated the antimicrobial activity of tea polyphenols against pathogens associated with endodontic infections.^12,15,16,30-31^ Cinnamon oil, thymol, carvacrol, cinnamaldehyde, and eugenol are among the natural plants and compounds that have been shown to have antimicrobial activity against endodontic pathogens, including *A. israelii*.^31^ A previous study has shown that tea polyphenols, particularly EGCG and theaflavins, efficiently inhibit the growth of *F. nucleatum*.^30^ Additionally, EGCG has also been shown to suppress the expression and activities of virulence factors of *S. mutans* associated with acidogenicity and aciduricity, making it potentially attractive anti-caries agent.^16^ However, different from our results, a previous study reported that EGCG was able to inhibit the growth or eliminate *E. faecalis* at low concentrations (5 and 20 µM, respectively) in 24h.^15^

In this study, the combination of polyphenol (EGCG) with an antibiotic (FOSFO) demonstrated synergism against all bacterial species, with a FIC index ranging from 0.35 to 0.5. This result is consistent with a previous study that showed that EGCG can increase the activity of β-lactams, likely due to the fact that both EGCG and β-lactams directly or indirectly attack the same site, peptidoglycan, on the cell wall^19^. This direct binding with peptidoglycan may also explain the synergism observed against methicillin resistant Staphylococcus aureus (MRSA) strains, as EGCG may induce damage to the bacterial cell wall and interfere with its biosynthesis.^19,32^

The antibacterial mechanism of EGCG involves damaging bacterial cell membranes, inhibiting the bacteria’s ability to adhere to the host cells, and downregulating enzymes involved in fatty acid biosynthesis to reduce the production of toxic metabolites.^32^ EGCG has also been shown to have a synergistic effect with β-lactams against methicillin-resistant strains of Staphylococcus aureus (MRSA) by downregulating PBP2a expression and upregulating lytM and lgrA expression. This results in increased secretion of autolytic enzymes, increased mucopeptide hydrolysis, and synergism with β-lactam antibiotics in inhibiting bacterial cell wall synthesis.^32^

In addition to ECGC, fosfomycin was also used in the present study as an antimicrobial agent. Fosfomycin interferes with the first stage of bacterial cell wall biosynthesis, specifically the formation of the peptidoglycan precursor UDP N-acetylmuramic acid (UDPMurNAc), which is linked to reduced bacterial adhesion to epithelial cells.^22^ Clinically, fosfomycin in combination with daptomycin, vancomycin, rifampin, and tigecycline has been shown to significantly reduce and modify the structure of *Staphylococcus aureus* (MRSA) and *Enterococcus faecalis* biofilms.^33,34^

In this study, the combination of EGCG and FOSFO at the highest concentration showed the lowest results in cell viability, yet still remained above 80%, demonstrating cytocompatibility. This is a crucial property for any compound recommended for endodontic treatment, particularly in young permanent teeth with incomplete root formation. EGCG has been shown to promote the differentiation and proliferation of pulp cells in collagen scaffolds at a concentration of 10 µM.^35^ In a recent study, EGCG up to 25 µM was not cytotoxic to stem cells of apical papilla and increased the expression of mineralization markers in the presence of mineralizing agents.^36^ Fosfomycin, similar to other penicillins such as amoxicillin, inhibits peptidoglycan biosynthesis, which results only in bacterial death. A previous *in vitro* study showed that poly-L-lactide acid (PLLA) synthetic polymer associated with several antimicrobials, including amoxicillin, inhibited *Porphyromonas gingivalis* and *Streptococcus pyogenes* growth without causing toxicity to fibroblasts.^37^

The antimicrobial effect of EGCG alone or in combination with FOSFO was evaluated at concentrations of 10× MIC /10× FIC for each species separately on monospecies biofilms or 10× the highest MIC/10× the highest FIC for multispecies biofilms, considering that microbial cells within biofilms are known to exhibit 10-1000 times more antibiotic resistance than planktonic cells^38^. In addition, in the present study, EGCG and CHX were considered controls, considering their application for clinical purposes, as proposed by other studies.^39,40^ Fosfomycin was not considered a control in these experiments since the combination of EGCG and fosfomycin proved to be synergistic, and the two compounds were considered as one antimicrobial agent. Besides, the use of conventional antibiotics is not recommended for biofilm control since they are unable to completely eradicate bacterial cells and may lead to drug resistance, contributing to chronicity of infections.^41^

The present study evaluated the effect of EGCG and FOSFO on monospecies biofilms, with *E. faecalis *and *A. israelii *being the most resistant to the treatments, although all biofilms were affected by the antimicrobial agents tested. Studies have indicated that *E. faecalis* is associated with cases of unsuccessful endodontic treatment and persistence of periradicular diseases.^6,8^ Lee and Tan^15^ (2015) reported that EGCG eliminated 7-day *E. faecalis* biofilms in dentin discs at a concentration of 500 µg. The authors also demonstrated that EGCG at sub-MIC significantly decreased the expression of virulence traits of *E. faecalis*, such as gelatinase, collagen-binding antigen, cytolysins, and proteases that are related to colonization, survival, and persistence of *E. faecalis* in the root canal.^15^ Other studies showed that EGCG was also able to inhibit the formation of *F. nucleatum *biofilm^30^and suppress glycosyltransferases genes in *S. mutans*, disrupting the initial attachment of *S. mutans* and, thus, the formation of mature biofilms.^16^ Some experimental studies (*in vitro *and biofilm infection models) showed that the combination of fosfomycin with other antimicrobial agents not only reduces or eradicates clinically significant bacteria from biofilms, but also modifies the biofilm structure.^33,34^ This study showed that the effect of EGCG in combination with fosfomycin in biofilms was higher in comparison to EGCG alone, possibly due to the combination of different mechanisms of action on the bacteria tested.

In multispecies biofilms, the combination of EGCG and fosfomycin (at 10× the highest FIC – 0.62 mg/mL EGCG and 0.078 mg/mL FOSFO) was found to be similar or superior to EGCG alone at a high concentration of 2.5 mg/mL. Bacterial counts were significantly reduced in both polystyrene microplates and dentin tubules when treated with the compounds and controls. Additionally, SEM images revealed that the association of EGCG and FOSFO affected the biofilm organization and extracellular matrix deposition. SEM images was also showed a predominance of cocci for *A. israelii* and *F. nucleatum, *despite providing all the *in vitro* conditions for their growth, including culture media and anaerobic conditions. This finding confirms that both *S. mutans *and *E. faecalis *grow faster than other rod-shaped bacilli, as observed in clinical conditions.^42-44^ As revised by Prada, et al.^42^ (2019), despite some heterogeneous results regarding the mostly prevalent pathogen found in teeth with endodontic failures are found, some bacterial species or gender of bacteria are prevalent in endodontic infections. Firmicutes (examples: streptococci and enterococci) and Bacteroidetes are the most abundant phyla detected in root canal infections, followed by Actinobacteria and Fusobacteria, regardless of the type of endodontic infections.^43^ Pinheiro, et al.^44^ (2003) observed that *Enteroccocus faecalis* was the most prevalent bacteria (45.8%) found in teeth with secondary infections, followed by *Streptococcus* (30%), *Actinomyces* (13.3%), *Fusobacterium* (6.7%), *Lactobacillus* (6.7%) *spp.*, and others*.* No study evaluating the effect of polyphenols combined with antibiotics in multispecies biofilms were found to compare with our results. However, a previous study observed that the combination of curcumin and EGCG was more effective in inhibiting the growth of multispecies biofilm (with nine Gram-negative bacteria) and in reducing polysaccharides content when compared with curcumin or EGCG alone.^45^ The authors also demonstrated that the combination of compounds tremendously reduced the biofilm thickness by confocal microscopy.^45^ In another study, polyphenols were tested on multispecies biofilms formed with *Streptococcus mitis, F. nucleatum*, *P. gingivalis, *and *Agregatibacter actinomycetemcomitans,* and it was observed that curcumin altered the architecture of mature multispecies biofilms and reduced their metabolic activity.^46^

Additional effects of EGCG may be expected in the root dentin and apical area. Studies have shown that byproducts of both root canal sealers and bacteria related to endodontic infections can activate proMMP-2 and -9, which could be lead to degradation of collagen fibrils within resin-bonded dentin interfaces.^47^ MMPs have also been also correlated with periapical bone resorption and apical periodontitis.^18^ EGCG has demonstrated the ability to inhibit root derived MMPs in a concentration dependent manner, increasing its effect from 200 to 600 µg/mL.^49^ In a rat model of induced apical periodontitis, EGCG significantly reduced periapical lesions as observed in radiography and histopathology analysis, although MMP activity was not evaluated in the study.^48^ However, there is limited clinical evidence on the effect of root therapy on MMP levels.^49^ Further studies should be conducted to evaluate the MMP inhibition by EGCG and EGCG+FOSFO.

## Conclusion

In this study, although all the conditions for bacterial survival (culture media and anaerobic environment) were maintained before preparation for confocal analysis, bacterial counts were not checked after the treatment. We used LIVE/DEAD^®^ stain which contains SYTO 9^®^ dye to stain live bacteria and propidium iodine dye to stain dead bacteria to identify total viable bacteria from dentin tubules, but individual bacterial counts were not achieved. Bacterial recovery from dentin chips by shaving dentin layers surrounding the root canal with dental burs is being considered for further studies. Considering the limitations of this experimental *in vitro* study, the results suggest that EGCG and fosfomycin demonstrated synergistic effect against bacteria of endodontic interest under planktonic and biofilm conditions without affecting cell viability. This suggests that the association of these compounds has potential as a medication for endodontic purposes.
