# ERRATUM

**DOI:** 10.1590/1678-7757-2023er001

**Published:** 2023-04-28

Due to a publishing error the article: “Synergistic antimicrobial potential of EGCG and fosfomycin against biofilms associated with endodontic infections”, published at Journal of Applied Oral Science 31(e-20220282):1-13. doi: 10.1590/1678-7757-2022-0282 was printed with the following error:

Where it reads:

Anuradha Prakki University of Toronto, Faculty of Dentistry - Dental Research Institute - Toronto - ON - Canada. Phone: +1 416-864-8238 e-mail: a.prakki@cirurgião-dentista

The sentence should read:

Anuradha Prakki - University of Toronto - Faculty of Dentistry – Dental Research Institute – Toronto – ON – Canada - Phone: +1 416-864-8238 - e-mail: a.prakki@dentistry.utoronto.ca

